# How dispersal rates depend on the prey capture strategy: A case study of Georgia's spiders

**DOI:** 10.1002/ece3.11372

**Published:** 2024-05-12

**Authors:** David Tarkhnishvili, Armen Seropian, Christoph Erhardt, Nino Kachlishvili, Hans‐Joachim Krammer, Nils Hein

**Affiliations:** ^1^ Institute of Ecology Ilia State University Tbilisi Georgia; ^2^ LIB – Leibniz Institute for the Analysis of Biodiversity Change, Biodiversity Center Zoological Research Museum Alexander Koenig Bonn Germany

**Keywords:** Araneae, DNA barcoding, glacial refugia, isolation‐by‐distance, molecular divergence rates, postglacial expansion

## Abstract

Large‐scale barcoding projects help to aggregate information on genetic variability of multiple species throughout their ranges. Comparing DNA sequences of both non‐conspecific and conspecific individuals from distant parts of their ranges helps to compare level of genetic isolation‐by‐distance patterns in different species and adaptive types. We compared mitochondrial CO1 gene sequences of 223 spiders from Georgia (Caucasus), representing 124 species and eight families, with 3097 homological sequences from spiders mostly from Europe, but also from other parts of the World. In most families, a significant isolation‐by distance pattern was observed on family level. On species level, a significant isolation‐by‐distance was observed in 40 species, although this low proportion is most likely related to a lack of data. Simultaneously, remarkable differences in spatial structure were shown for different species. Although the majority of the studied species have a broad western Palearctic range, web‐building spiders from families Araneidae, Theridiidae, and Linyphiidae are less isolated spatially than flower spiders (Thomisidae), jumping spiders (Salticidae), wolf spiders (Lycosidae), sac spiders (Clubionidae), and ground spiders (Gnaphosidae). This pattern is related with more common ballooning in web building than in actively hunting spiders, which commonly remain isolated since preglacial time. Ground spiders build the most isolated populations in the Caucasus.

## Graphical abstract

This article compares genetic differentiation within 124 Eurasian species from eight families of spiders, based on the barcoding data. The conspecific web‐building spiders from the Caucasus and the rest of Eurasia are significantly less differentiated than the actively hinting species, although isolation‐by‐distance pattern presents in at least half of the species. We conclude that prey capture strategy significantly impacts dispersal ability in spiders.

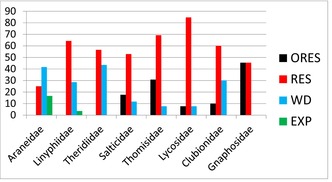

## 1 INTRODUCTION

Pleistocene glacial cycles shaped the current biodiversity of nontropical areas. During long‐lasting glacial waves, most of the biological species have been extinct from most part of the land in the northern hemisphere: Climatic conditions there became extreme, and only few animals and plants could survive in these areas, similar to what we see now in subarctic and arctic ecosystems (Abbott et al., 2000; Hewitt, 1996; Hofreiter & Stewart, 2009). Others were captured in isolated refugia, where the climate remained reasonably mild and humid (Bennet & Provan, 2008; Feliner, 2011; Fu & Wen, 2023; Hewitt, 1996; Tarkhnishvili et al., 2012). In these refugia, they continued to evolve, and populations from isolated landscape patches diverged genetically and phenotypically (Avise, 2000; Hewitt, 2001; Mutanen et al., 2012; Petit et al., 2003; Vila et al., 2005). During shorter interglacial periods, lasting 10–20 Kyr, populations of living organisms expanded from individual refugia and admixed with animals and plants that inhabited remote areas. As a result, the observed biodiversity of the areas that endured glacial waves is shaped by immigrants from former refugia, expanded during the last 10–15 Kyr. Current biodiversity in or around glacial refugia comprises the populations that survived locally and those expanded from the refugia located in different geographic areas.

Obviously, different organisms disperse at different rates, depending on the reproduction speed and phenotypic features of the organisms themselves. Birds and butterflies, most likely, expand faster than snails or amphibians. As a result, bird faunas of individual geographic regions are more speciose than the amphibian faunas (Crnobrnja‐Isailovic, 2007; Veech & Crist, 2007), but simultaneously, proportion of the local endemics among continental birds is lower than the proportion of the endemics in amphibians or snails. We also expect that the dispersal rates differ for different evolutionary lineages within the same major taxon.

For the geographic areas within or close to glacial refugia, such as the Caucasus Ecoregion (Tarkhnishvili, 2014; Zazanashvili et al., 2004), one can expect that the taxonomic groups with the highest dispersal ability would have the highest proportion of species that immigrated in the postglacial time from other areas and, hence, the lowest proportion of regional endemics. It is also possible that species diversity will be the highest for such groups. Their alpha diversity should be high, but beta diversity (Socolar et al., 2016) relatively low. Conversely, the taxa with low dispersal ability may have a higher proportion of endemics and less species diversity but relatively high beta diversity. Analysis of patterns of species diversity in this context helps us to better understand how the biodiversity of individual regions is formed, to identify taxonomic groups whose presence is limited with specific geographic areas, and hence better concentrate conservation efforts.

Spiders (Araneae) are among the most diverse noninsect animal taxa. The newest checklists of Georgian spiders (Otto, 2023; Seropian, Bulbulashvili, et al., 2023; Seropian, Otto, & Bulbulashvili, 2023) count 756 species from 44 families. We have selected 124 species of Georgian spiders from eight families, barcoded by sequencing CO1 mitochondrial gene, and hence comparable with the conspecific individuals from other parts of the World. It gave us a unique opportunity to analyze the level of genetic isolation of spiders from families with different prey capture strategies and estimate the dispersal abilities on the species and family levels.

Spiders may disperse in different ways. Besides walking on the ground or plant surface, in a way similar to other nonflying arthropods, they use a unique way of dispersal called ballooning (Blandenier & Fürst, 1998; Hormiga, 2002; Macías‐Hernández et al., 2020; Simonneau et al., 2016). Ballooning includes releasing a silken thread from the abdomen, which is then transported by air masses, lifting, and dragging the spider. The ballooning individual is then wallowing air currents and do not control the direction and distance of displacement. The ballooning behavior and aerodynamics of ballooning is described in detail by Zhao et al. (2017). The most of spider ballooners displace for a few hundred meters, although individual spiders can move for hundreds of kilometers (Blandenier, 2009). In some occasions, ballooning can move spiders on the distance of 16,000 km (Hormiga, 2002). Zhao et al. (2017) suggest that the factors determining the distance of displacement of ballooning spiders depends on the weight of a spider, strength and local dynamics of wind, as well as on electrostatic forces.

Irrespectively of the factors considered by aerodynamic models, the ability to produce more silk threads could be a decisive factor determining the dispersal ability of individual species and taxonomic groups, which is by the way highly variable (Blandenier, 2009; Domènech et al., 2022). This research aims to answer two research questions related to the influence of dispersal ability dependent on the adaptive phenotype on the species and genetic diversity. One of our hypothesis was that spiders, which more commonly produce hunting webs, more commonly use ballooning for dispersal, and hence should be less structured genetically at a large geographic scale than those that do not produce webs for hunting. This hypothesis is in line with the results of studies on a finer geographic scale (Domènech et al., 2022). The second question was that, within the studied area, the families with higher dispersal ability are more speciose than those with limited dispersal ability because their local faunas are composed of both species that survived the Last Glacial Maximum (LGM) locally and those that expanded into the region in postglacial time. Finally, we hypothesized a discrepancy between the formal taxonomy and the level of genetic differentiation, assuming that some species diverge phenotypically faster than others.

## 2 MATERIALS AND METHODS

### 2.1 Sampling and species identification

Within the framework of the Caucasus Barcode of Life project (CaBOL), 232 individual spiders representing 124 species from eight families (Araneidae, Linyphiidae, Theridiidae, Salticidae, Thomisidae, Lycosidae, Clubionidae, and Gnaphosidae) were collected, identified, barcoded, and stored in the collection at the Institute of Zoology, Ilia State University, Tbilisi, Georgia, and the Museum Koenig, Bonn, Germany. Morphological identification was done using literature sources on Caucasian spiders (see list in Otto, 2023) as well as Nentwig et al. (2023), WSC (2024), and sources listed therein. A Zeiss Stemi 508 Stereo Microscope with 8:1 Zoom and a Zeiss Apo 1.5x FWD 53 mm front lens were used for species identification (Seropian, Bulbulashvili, et al., 2023; Seropian, Otto, & Bulbulashvili, 2023). Table S1 shows species' affiliation, collection number, barcoding number, and geographic location of each individual. Table 1 shows species diversity in each of the studied families in Georgia and the number of species and genera used in this study. Not surprisingly, the highest number of the barcoded genera and species is from more diverse families.

**TABLE 1:** Species and genera diversity of eight spider families in Georgia (according to Seropian, Bulbulashvili, et al., 2023; Seropian, Otto, & Bulbulashvili, 2023) and the number of species used in this study.

Families	Genera in Georgia	Species in Georgia	Genera in this study	Species in this study
Araneidae	19	54	9	11
Linyphiidae	75	137	22	28
Theridiidae	31	70	18	22
Salticidae	44	102	9	17
Thomisidae	18	64	7	13
Lycosidae	15	83	6	12
Gnaphosidae	18	54	9	11
Clubionidae	2	15	1	10
Total	222	579	81	124

### 2.2 DNA extraction and sequencing

The specimens were preserved in 96% ethanol and stored in the freezer at −20°C. DNA was isolated from spider legs or from the whole specimen. The legs were used in spiders, whose body length was over 1 cm. For spiders whose length was ≤1 cm, the whole organism was used in the lysis process. After overnight lysis, the specimens were replaced and preserved in 96% ethanol. DNA purification was processed with a quick‐DNA mini prep plus kit (for specimens over 1 cm) and a quick‐DNA micro prep plus kit (for specimens smaller than 1 cm) (Zymo Research, D4074, and D4069). DNA barcode region of mitochondrial Cytochrome Oxidase subunit 1 (CO1) was amplified using the primer pair LCO1490‐JJ and HCO2198‐JJ (Astrin & Stüben, 2008). Protocol for PCR reaction was created according to Astrin et al. (2016). A total 20 μL volume PCR reaction was containing 1 μL (10 pmol/μL) of each primer, 0.4 μL Promega GoTaq DNA polymerase, 2 μL of 25 mM MgCl_2_, 4 μL of 5× Promega green buffer, 0.8 μL of 10 mM dNTPs mix from Thermo Fisher Scientific and 3 μL spider DNA, with the following thermal conditions: 15 cycles (touch down) of denaturation at 94°C for 30 s, annealing at 55°C for 60 s (−1°C per cycle), and extension at 72°C for 90 s, followed by another set of 25 cycles: denaturation 94°C for 35 s, annealing 45°C 60 s, and extension of 72°C for 90 s. PCR products were sent for bidirectional Sanger sequencing to BGI company (Hong Kong, China). AB files were processed in Geneious Prime 2022 1.1. Extracted DNA was deposited in the scientific collection of Ilia State University, Tbilisi, Georgia, and the aliquots will be deposited at the Leibniz Institute for the Analysis of Biodiversity Change (LIB, Biobank, former Museum Koenig, Bonn, Germany), and the sequences (658 bp fragment of CO1) were submitted to the Barcode of Life Data System (BOLD) database.

### 2.3 The identification of the closest genetic relatives

The database BOLD (Ratnasingham & Hebert, 2007) was used for downloading sequences of the barcoded individuals that belong to the same species from different parts of the World. For downloading the sequences close to those of the studied specimens, CE developed a python program CaBOL_BOLD, which automatically compares a fasta file with the online Barcode of Life Database (BOLD) and downloads the hits and the associated metadata. Then, the distance between source countries from this metadata and the CaBOL specimen source country is calculated automatically and written into a preformatted Excel result sheet alongside all the metadata for further scientific analysis. The detailed description/access to the program see in Appendix 1. The analysis of the downloaded sequences showed the proportion of substitutions between conspecific individuals varying between 0% and 7.2% (below 3.8% in 95% of cases, which is substantially higher than calculated for the Central European populations—Astrin et al., 2016). In the further analyses, we used all conspecific sequences with genetic differences not exceeding 7.72%.

### 2.4 Analysis of factors affecting genetic closeness of the individuals

We applied several statistical methods for exploring the factors that correlate with the genetic distance of Georgian individuals from the spiders collected in other parts of Eurasia and North Africa.

In order to test whether geographic distance affects the genetic distance between the studied individuals and whether the effect is species‐specific and family‐specific, we applied a linear mixed model (Bolker, 2015). We ran the model twice, with families and with the species as a nominal predictor.

We used linear regression analysis to infer the correlation between the geographic and genetic distances, applying it to the entire dataset, individual families, and species of spiders. For the whole dataset, we saved unstandardized residuals from the regression line to establish representatives of which spider families increase their genetic distance with geographic distance slower and, conversely, faster than the average. We also calculated the regression lines and the residuals separately for each of the eight studied families for identifying the species with slower and faster dispersal rates than the family on average. For testing significance of correlation between geographic closeness and genetic similarity, we used only those species which had six or more closely related sequences in BOLD database.

Some species are found far beyond the western Eurasia, for instance, in North America. However, the North American samples may shadow general patterns of isolation‐by‐distance, especially if one considers their possible recent relocation by humans (Dondale & Redner, 1990; Emerton, 1902; Ivie, 1969; Keyserling, 1886; Vetter et al., 2014; WSC, 2024). For this reason, we did not include these samples in the analyses described above, but discuss them separately.

### 2.5 Interpretation of the molecular clock

Papadopoulou et al. (2010) inferred the average divergence rates for insect CO1 (0.013 per million years). We assume similar divergence rates in spiders. Under this assumption, we simulated the mutation rates under the Poisson distribution model:

$$f\left(k;\lambda \right)=\left(\lambda^{k*}e^{-\lambda }\right)/k!$$

where *k* is the number of substitutions for the fragment with length 658 bp, *λ* is the average number of mutations per year (0.000000013), and *e* is the Euler number. Under these assumptions, the lower 95% limit for HPD (high probability density) distribution of divergence time for more than two substitutions is ca. 40 Kyr. Hence, if two sequences from distant geographic regions differ in more than two substitutions, their isolation lasts from the pre‐Holocene time, and postglacial expansion, including that caused by humans, is unlikely. If two sequences from distant geographic regions differ in 14 or more substitutions, the lower 95% HPD limit exceeds 2.6 Mya, which is the conventional upper boundary of Pleistocene glaciations (Lourens et al., 2004); hence, the respective populations are isolated from preglacial time. Unfortunately, our model does not rule out the probability that the sequences, which do not differ at all, may still belong to individuals isolated from pre‐Holocene time; the upper 95% HPD limit is ca. 400–600 Kyr. However, the probability that these species did not have gene flow among the distant geographic regions for at least 80 Kyr, exceeds 50%. Assuming that the expansion during a glacial period is unlikely, we can suggest that their expansion during Holocene is more likely than that in previous interglacial periods. The described model provides simple albeit more conservative estimates of the isolation time than the boundaries inferred from the developed Bayesian algorithms such as BEAST (Drummond & Rambaut, 2007). The calculations are presented in Appendix 2.

All calculations were conducted with Excel v.14 (Microsoft Corporation, 2018) and IBM SPSS statistics 23.1 (IBM Corp., 2015).

## 3 RESULTS

### 3.1 Factors significantly affecting genetic distance

Mixed model showed a highly significant influence of the species, the family, and geographic distance on the genetic similarity between the samples for the entire dataset. It also suggested different influences of the geographic distance on the genetic similarity between the individual species (Table 2). The negative correlation between geography and genetic closeness was weak but highly significant for the entire dataset and for six out of eight studied families, except wolf spiders (Lycosidae) and sac spiders (Clubionidae); Table 3.

**TABLE 2:** The output of the LMM analyses: The effect of species (same or different), families (same or different), geographic distance (km between the locations/countries), and join effect of species/distance and family/distance on the genetic distance between the samples (proportion of differences in a studied fragment of CO1 gene).

Source	Numerator df	Denominator df	*F*	Significance
Intercept	1	3077	37.4348	<.0001
Species	112	3077	4.455	<.0001
Distance	1	3077	50.818	<.0001
Species × Distance	110	3077	7.756	<.0001
Intercept	1	3304	32.28565	<.0001
Family	6	3304	8.896	<.0001
Distance	1	3304	74.001	<.0001
Family × Distance	6	3304	10.108	<.0001

*Note*: Repeated variable—an individual from the CaBOL database.

**TABLE 3:** Pearson correlation coefficients between geographic closeness and genetic similarity for individual spider families and the entire dataset.

Family	*N*	Correlation coefficient	*p*
Araneidae	307	−.154	.007
Linyphiidae	1291	−.239	<.0001
Theridiidae	557	−.160	.0002
Salticidae	203	−.313	<.0001
Thomisidae	218	−.361	<.0001
Lycosidae	312	.066	.246
Gnaphosidae	153	−.250	.002
Clubionidae	280	−.025	.680
All studied families	3041	−.140	<.0001

Of 124 studied species, 102 had six or more similar sequences downloaded from the BOLD database. Among those, in 40 species, negative correlation between the geographic distance and genetic closeness was significant (*p* < .05), and five (*Diplostyla concolor*, Linyphiidae; *Phylloneta impressa*, Theridiidae; *Thomisus onustus*, *Xysticus acerbus*, and *X. luctator*; Thomisidae) had a significant positive correlation. In the latter five species, the genetic distances between Georgian and Eastern and Central European samples were higher than that between the Georgian samples and those from France. For most individual species, the correlation was, however, insignificant, probably due to insufficient sample size (Table S2).

### 3.2 Proportion of endemic, resident, and expanding species

**FIGURE 1:**
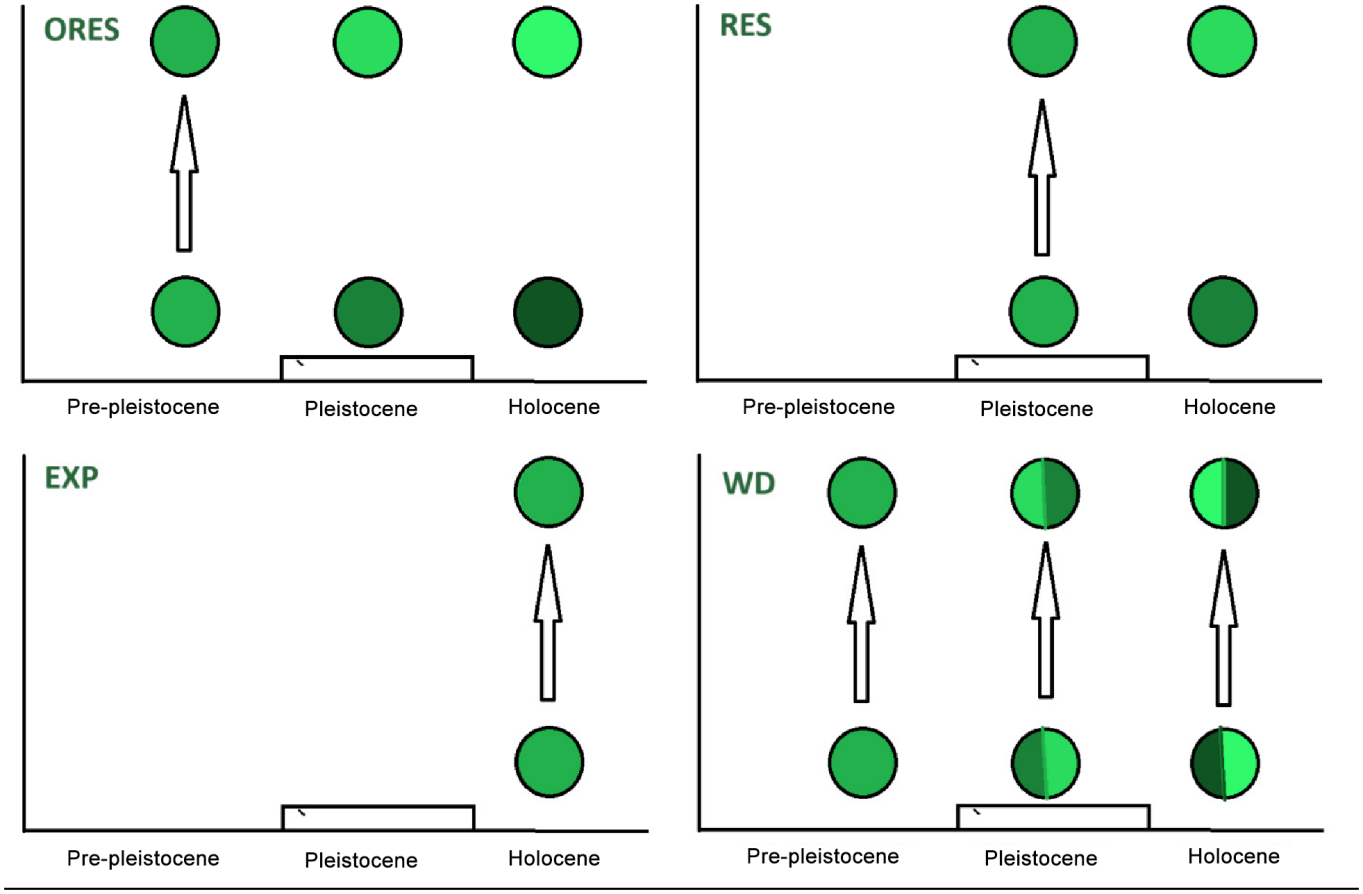
Four types of genetic structure on geographic scale. ORES—species that expanded in preglacial time through the current range and locked in different regions since Pleistocene glacial cycles. RES—species that expanded during Pleistocene interglacial periods but had no gene flow in Holocene. EXP—species expanded throughout their current range in Holocene. WD—species that formed their current range in Pliocene or Pleistocene, but gene flow between distant geographic populations continues until now. Contrasting colors indicate the level of genetic differences.

Among the studied species, only three have a limited regional distribution. The ranges of *Clubiona caucasica*, *Ozyptila tricoloripes* (Thomisidae), and *Centromerus minor* (Linyphiidae) are limited to the Caucasus and adjacent parts of Asia Minor and southern Russia (WSC, 2024). The studied species can be divided into four categories: (1) “old residents” (ORES), whose populations from Georgia and European countries are separated since preglacial time; (2) “residents” (RES), whose populations from Georgia and European countries are isolated at least since before 600 Kyr (>2 substitutions); (3) “expanders” (EXP), those species which populations show no genetic differences throughout the Caucasus and Europe; and (4) “wanderers” (WD), those species whose regional populations show admixture of different and coinciding haplotypes, and hence, are expanding during each consecutive interglacial, followed by isolation periods during glacial waves (Figure 1).

In Table 4, spider species are listed, whose sequences from outside Georgia are either identical or more or less different, suggesting the time of isolation before the Holocene, or before the Pleistocene Ice Age. Georgian populations of 13 species with a broad Eurasian or West Eurasian distribution are isolated from conspecific spiders from Europe at least since preglacial time (over 2.5 Mya; ORES). Those are *Callilepis nocturna*, *Civizelotes caucasius*, *Gnaphosa steppica*, *Haplodrassus signifier*, *Haplodrassus silvestris* (Gnaphosidae), *Alopecosa albofasciata* (Lycosidae), *Attulus rupicola*, *Pellenes seriatus*, *Phlegra cinereofasciata* (Salticidae), *Ozyptila atomaria*, *Ozyptila claveata*, *Xysticus luctator* (Thomisidae), and *Clubiona lutescens* (Clubionidae). This list does not include any species of the true web‐building spiders, in spite of high species diversity of the three respective families.

**TABLE 4:** Spiders of eight families, which do not show gene flow since at least pre‐Holocene time (RES), and those which show Holocene gene flow between Georgian populations and those from distant regions (WD).

Groups	RES	WD
Araneidae	*Argiope lobata*, *Cercidia prominens*, *Leviellus caspicus* ^b^	*Aculipera ceropegia*, *Araneus quadratus*, *Larinoides cornutus*, *Nuctenea umbratica*
Linyphidae	*Acartauchenius scurrilis*, *Bolyphantes alticeps*, *Centromerus minor* ^a^, *Centromerus sylvaticus*, *Ceratinella brevia*, *Diplocephalus latifrons*, *Gonatium rubens*, *Megalepthyphantes pseudocollinus*, *Metopobactrus prominulus*, *Neriene clathrata*, *Palliduphantes khobarum* ^b^, *Sintula retroversus*, *Tallusia experta*, *Tenuiphantes mengei*, *Tenuiphantes tenius*, *Walckenaeria altipes*, *Walckenaeria carniculans*, *Walckenaeria monoceros*	*Centromerus minor*, *Diplostyla concolor*, *Erigone dentipalpis*, *Frontinella frutetorum*, *Microneta varia*, *Porrhomma microphtalmum*, *Styloctetor romanus*, *Tiso vargans*
Theridiidae	*Anelosimus vittatus*, *Coscinida tibialis* ^a^, *Crustulina guttata*, *Crustulina sticta*, *Cryptachea iparia*, *Dipoena torva*, *Enoplognatha serratosignata*, *Heterotheridion nigrovariegatum*, *Kochiura aulica*, *Phylloneta impressa*, *Robertus arundineti*, *Theridion varians*, *Yaginumena maculosa* ^b^	*Enoplognatha thoracica*, *Episinus truncates*, *Euryopis episinoides*, *Latrodectus tredecimguttatus*, *Neottiura suaveolens*, *Neottiura uncinata*, *Parasteatoda lunata*, *Platnickina tincta*, *Robertus arundineti*, *Simitidion simile*
Salticidae	*Attulus penicilloides*, *Attulus saltator*, *Cyrba ocellata* ^b^, *Dendryphantes rudis*, *Heliophanus lineiventris*, *Myrmarachne formicaria*, *Pellenes diagonalis* ^a^, *Pseudeuophrys erratica*, *Pseudicius encarpatus*	*Pellenes nigrociliatus*, *Saitis tauricus*
Thomisidae	*Bassaniodes pseudorectilineus* ^a^, *Diaea livens*, *Ebrechtella tricuspidata*, *Ozyptila tricoloripes* ^b^, *Pistius truncates*, *Psammitis ninnii*, *Thomisus onustus*, *Xysticus kempeleni*, *Xysticus marmoratus*	*Xysticus acerbus*
Lycosidae	*Alopecosa inquilina*, *Alopecosa pulverulenta*, *Alopecosa sulzeri*, *Alopecosa taeniopus* ^a^, *Arctosa tbilisiensis* ^a^, *Pardosa amentata*, *Pardosa nebulosa*, *Piratula latitans*, *Trochosa* sp.^b^, *Trochosa terricola*, *Wadicosa fidelis* ^b^	*Piratula latitans*
Gnaphosidae	*Drassodes pubescens*, *Gnaphosa lucifuga*, *Micaria formicaria*, *Scotophaeus scutulatus*, *Zelotes longipes*	–
Clubionidae	*Clubiona frisia*, *Clubiona germanica*, *Clubiona neglecta*, *Clubiona pseudoneglecta*, *Clubonia pallidula*, *Clubonia phragmitis*	*Clubiona brevipes*

^a^ Species whose sequences, besides Georgia, were available only from neighboring Turkey, Iran, and Russian Caucasus; hence, the degree of genetic differentiation from European or Central/Southern Asian populations remains unknown.

^b^ Species, whose sequences were available only for Asian populations, from Israel and Kazakhstan to China, but not in Europe; hence, the degree of genetic differentiation from the European populations remains unknown.

**FIGURE 2:**
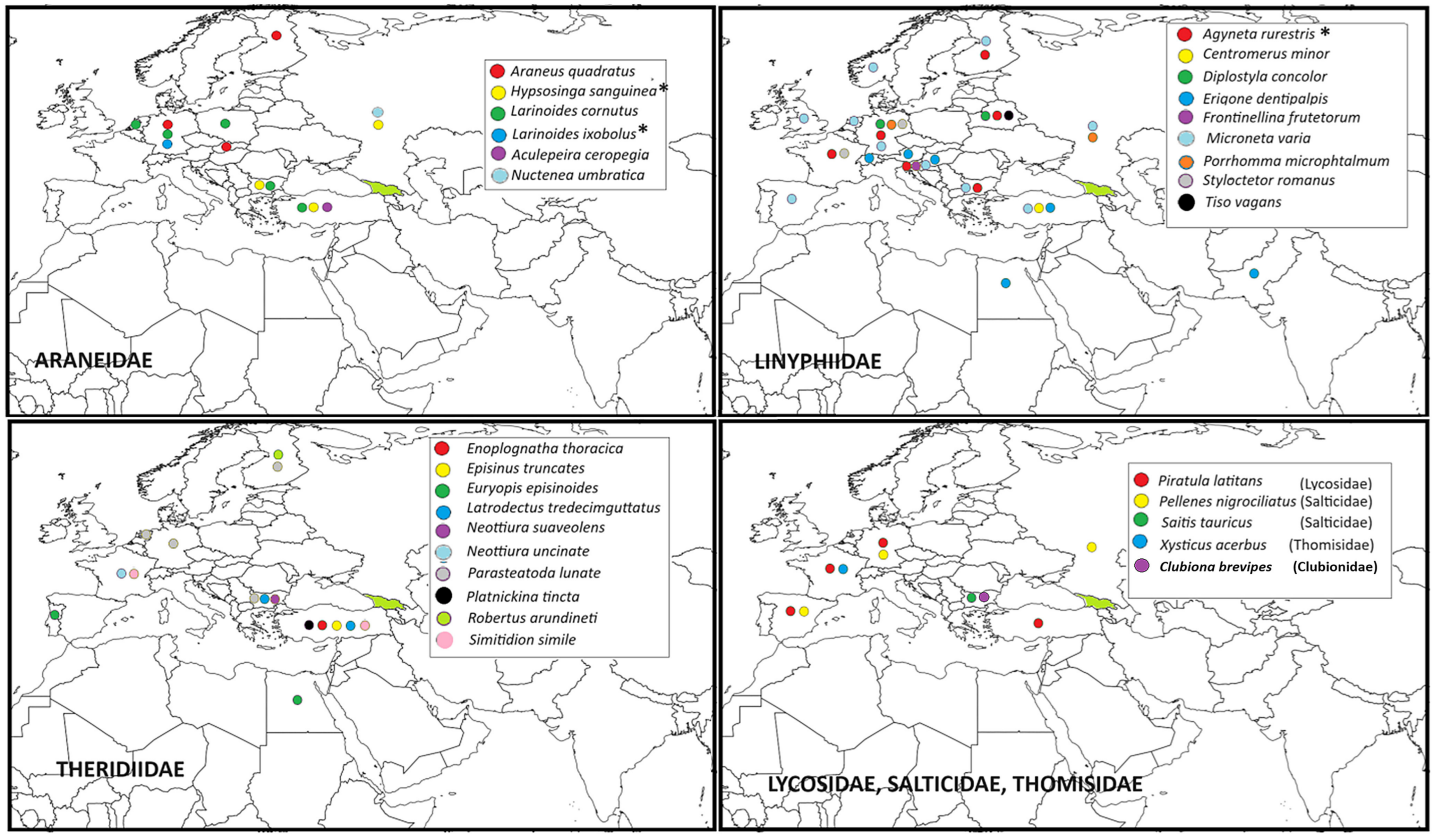
Species, whose populations from Europe, North Africa, or Southern Asia have identical CO1 sequences with the Georgian conspecific samples. Georgia marked with light green color. *Expanding species, only identical sequences were found irrespectively of the country of origin.

In contrast, “wandering” species, which have individuals with both identical and nonidentical haplotypes more or less far from the study area, are almost exclusively web‐building spiders (Table 4, Figure 2). Finally, there are only three “expanding species” (EXP), which show *only* identical haplotypes irrespective of the origin of sample: two from family Araneidae (*Hypsosinga sanguinea* and *Larinoides ixobolus*) and one from the family Linyphidae (*Agyneta rurestris*); remarkably, the latter species is known as the most frequent ballooner in Central Europe (Blandenier, 2009).

**FIGURE 3:**
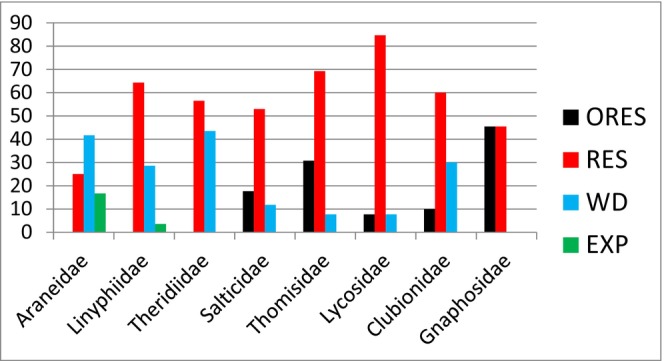
Percentage of “old residents” (ORES: more than 13 substitutions at 658 bp fragment of CO1 mitochondrial gene; species whose populations in Georgia are isolated since preglacial time), “residents” (RES: more than two substitutions, the populations are isolated at least since pre‐Holocene time); “wandering” (WD: identical sequences present in Georgia and other regions); and “expanding” (EXP; only identical sequences are found in samples from different countries).

The most of species from all families, however, belong to the residents, whose Georgian populations were isolated before Holocene (Table 4, Figure 3). Those include even the species which potentially might easily expand, for example, following flower trade routes, such as the widespread crab spider *T. onustus*.

Six species, *Parasteatoda tabulata* (Theridiidae), *D. concolor*, *Erigone dentipalpis*, *Tenuiphantes tenuis* (Linyphiidae), *Trochosa terricola* (Lycosidae), and *Clubiona pallulida* (Clubionidae), all with a broad western Eurasian distribution (WSC, 2024) were identical or almost identical (up to two substitutions) with the spider sequences from Canada; they obviously expanded to North America with human assistance, that is, are alien species there (Dondale & Redner, 1990; Emerton, 1902; Ivie, 1969; Keyserling, 1886; Levi, 1980; WSC, 2024). Besides these species, *Latrodectus tredecimguttatus* (Theridiidae), the European Black Widow, is likely an alien species in South Africa. Hence, 5% of species from our sample dispersed in distant regions of the World with human assistance. The sequences of these species from North America were excluded from the further statistical analyses, because they could shadow the isolation‐by‐distance patterns.

In general, in web‐building spider families (Araneidae, Linyphidae, and Theridiidae), there is a higher proportion of expanding and wandering species and less old isolates than in actively hunting species from families Salticidae, Thomisidae, Lycosidae, and Gnaphosidae (Figure 3).

### 3.3 Isolation‐by‐distance

Table 5 shows the parameters of the linear regression of genetic closeness on geographic distance. Salticidae had the most abrupt negative slope of the regression line, followed by Thomisidae, Gnaphosidae, Araneidae, Linyphiidae, Lycosidae, Theridiidae, and Clubionidae; the differences between most of the families were insignificant; however, in Salticidae and Thomisidae, the slope of the regression line is significantly (*p* < .05) more abrupt than in Araneidae, Gnaphosidae, Lycosidae, Theridiidae, and Clubionidae.

**TABLE 5:** The parameters of linear regression of genetic closeness of the samples on the geographic distance.

Family	Unstandardized coefficients	Standardized		*t*	Significance
	*B*	SE	*β*		
Araneidae					
Intercept	99.643	0.237		419.720	.0000000
Distance, km	−0.00027	9.765E‐05	−.154	−2.725	.0068051
Linyphiidae					
Intercept	99.420	0.055		1814.263	.0000000
Distance, km	−0.00020	2.304E‐05	−.239	−8.564	.0000000
Theridiidae					
Intercept	99.348	0.058		1711.172	.0000000
Distance, km	−0.00006	1.624E‐05	−.160	−3.809	.0001551
Salticidae					
Intercept	99.574	0.199		499.431	.0000000
Distance, km	−0.00040	8.575E‐05	−.313	−4.673	.0000054
Thomisidae					
Intercept	99.079	0.132		749.038	.0000000
Distance, km	−0.00035	6.139E‐05	−.361	−5.691	.0000000
Lycosidae					
Intercept	98.760	0.153		646.097	.0000000
Distance, km	0.00007	6.354E‐05	.066	1.161	.2464853
Gnaphosidae					
Intercept	98.010	0.245		400.260	.0000000
Distance, km	−0.00031	9.825E‐05	−.250	−3.170	.0018471
Clubionidae					
Intercept	99.048	0.139		712.506	.0000000
Distance, km	−0.00002	4.90E‐05	−.025	−0.413	.6800000

**FIGURE 4:**
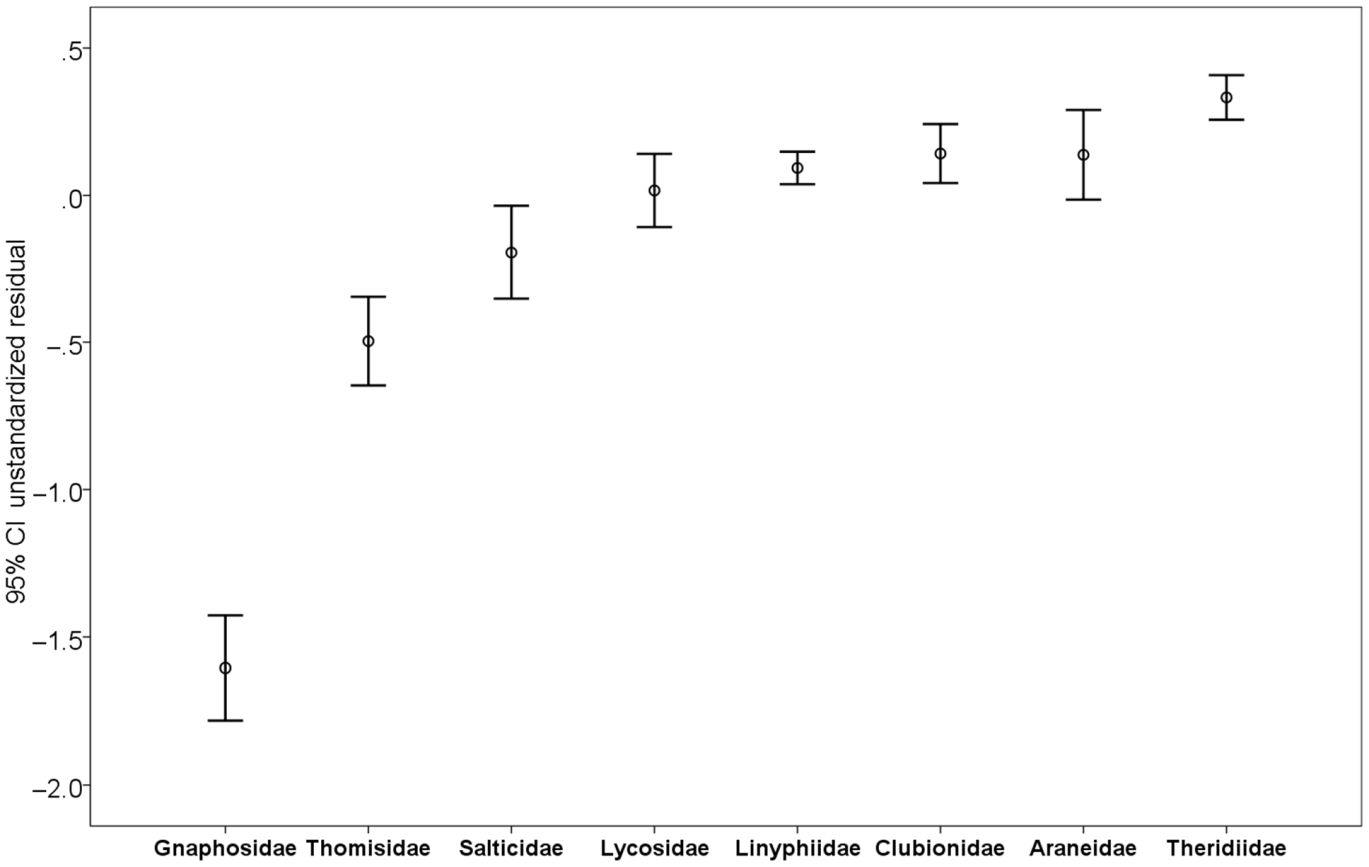
Average residuals from the regression line of genetic versus geographic distance and 95% confidence intervals from the overall regression line in the studied spider families.

The highest average residuals from the overall regression line were in families Theridiidae, Araneidae, Clubionidae, and Linyphiidae; the lowest—in Gnaphosidae and Thomisidae (Figure 4). In the families with the highest average values, the genetic distance increases the most rapidly with distance. The regression parameters for the individual families are shown in Table 5.

In spite of higher dispersal ability, the families of web‐building spiders are not more diverse than the families of active hunters. Jumping spiders (Salticidae) are the second more diverse family in Georgia, and they are more diverse than orb‐weavers (Araneidae). No significant correlation exists between the number of species or genera in a family and any of the measures presented in Tables 3, 4, 5. However, the Linyphiidae, which have the highest proportion of mitochondrial lineages shared between the Caucasus and Europe (36% of the studied species), have also the highest species diversity in Georgia.

## 4 DISCUSSION

Our study suggests that most of Georgia's spiders are locked geographically in the area, covering the Caucasus and Asia Minor, and did not have contact with the conspecific European populations since the last glacial cycle. Simultaneously, at least 28% of the species had gene exchange with the European populations in the Holocene. Spiders of the family Linyphiidae (sheet wavers) appear to have the highest dispersal capacities: 36% of the studied species have mitochondrial lineages shared between the Caucasus and Europe, suggesting that the populations from these geographic regions were never isolated from each other. Spiders of the families Araneidae (orb‐weaver spiders) and Theridiidae (tangle‐web spiders) are also easily dispersing throughout Western Eurasia. It is additionally supported by a weak, although significant correlation between the genetic similarity and geographic distance in Linyphiidae, Araneidae, and Theridiidae. Conversely, populations of ground spiders of the family Gnaphosidae, in spite of the absence of formally endemic species, appear to be isolated within the Caucasus Ecoregion, and their populations never exchanged genes with the conspecific populations from Europe since at least Last Glacial Maximum, and for almost half of the species—since pre‐Pleistocene time. All but one or two crab spiders (Thomisidae), Jumping spiders (Salticidae), sac spiders (Clubionidae), and wolf spiders (Lycosidae) from Georgia are also effectively isolated from the conspecific populations in Europe.

To explain these differences between the studied families of spiders, we should consider their adaptive morphology and ecology. Spiders of families Thomisidae, Salticidae, Gnaphosidae, Clubionidae, and Lycosidae do not build webs for catching prey, whereas those from the families Araneidae, Linyphiidae, and Theridiidae do (Cardoso et al., 2011; Fernández et al., 2014; Savory, 1928). In web‐building spiders, ballooning is a widespread dispersal mode for young specimens (Blandenier, 2009; Domènech et al., 2022), which can be transported by wind on a maximum distance of up to 16,000 km (Hormiga, 2002). Ballooning is considered a passive dispersal method and occasionally exists in actively hunting spiders as well, although it is not that widespread (Macías‐Hernández et al., 2020; Suter, 1999). Previous studies showed that ballooning spiders are often immature and rarely have a mass >1.2 mg (Greenstone et al., 1987; Richter, 1970). Macías‐Hernández et al., (2020) summarized ballooning frequency in 506 spider species from Iberian Peninsula and Macaronesia. They showed that all species of Araneidae and almost all Linyphiidae are frequent ballooners; 97% of Lycosidae, as well as all Theridiidae, Salticidae, and Thomisidae are occasional ballooners and, finally, all Gnaphosidae and Clubionidae are rare ballooners. This is in line with the long‐term study of ballooning spiders in Central Europe (Blandenier, 2009), who also identified Linyphiidae and Araneidae (but not Theridiidae) as the most common ballooners. Simonneau et al. (2016) studied ballooning spiders in agricultural landscapes of France. Linyphiidae was the predominant family (74% of the individuals studied), followed by Theridiidae and Araneidae. Although we did not conduct field study for estimating of ballooning frequency in different families, weak structure of the West Eurasian population in Theridiidae, similar to two other studied families of web‐building spiders, suggest importance of ballooning in this family as well, in line with findings of Simonneau et al. (2016). This is also concurrent with the suggestion of Bonte, Lens, et al. (2003) that well‐developed silk production in web‐building spiders increases probability to balloon.

We can conclude that ballooning is the key factor affecting expansion potential in spiders, in its turn associated with producing cobwebs; hence, cobweb building, which independently evolved in several clades of spiders (Fernández et al., 2014), is an important general adaptation, not only for saving energy while catching prey (Eberhard, 1990 for review) but also increasing dispersal rates on short (Botham et al., 2020), middle (Domènech et al., 2022), as well as on very large distances (this study), and hence, the effective size of spider populations.

We suggest this is a primary explanation of the differences between the two groups, “residential” and “expanding” families. It is more difficult to explain the differences between the four families of actively hunting spiders with a limited dispersal ability using ballooning. In the flower spiders and the jumping spiders, the excess of resident species correlates with the presence of isolation‐by‐distance pattern. It is not necessarily causing the emergence of endemic forms: A number of widespread species show little phenotypic differentiation within their broad ranges; however, this is concurrent with substantial genetic differences between the Caucasian and Central European populations. In contrast, in wolf spiders (Lycosidae) and sac spiders (Clubionidae), a very high level of residence is paralleled with a less clear pattern of isolation‐by‐distance. One possible explanation for this is active dispersal by “walking”: for instance, some wolf spiders of the genus *Pardosa* may walk several hundred meters per day along a simple, monotonous landscape (Bonte, Vandenbroecke, et al., 2003; Richter et al., 1971). As a result, the population structure of “walking” spiders is homogenous through large areas with continuous landscapes, such as the Caucasus through Anatolia; however, natural barriers such as the Bosporus effectively isolate them from the European populations. Besides, the distribution patterns differ among the species from the same family. In spite of the limited dispersal ability in freely hunting spiders, single species from each of the three families, Lycosidae, Thomisidae, and Salticidae, share CO1 haplotypes between the Georgian and Central European populations, suggesting recent gene exchange between these regions.

Therefore, we can identify three groups within the studied eight spider families: (1) Spiders with a high dispersal potential, probably related to the ballooning: Araneidae, Linyphiidae, and Theridiidae. The Western Eurasian populations of these spiders do not show a well‐expressed isolation‐by‐distance pattern and do not form effectively isolated geographic subpopulations in the Holocene. Gene flow occurs even between the populations separated by a geographic distance exceeding 2000 km. (2) Spiders with a low dispersal potential, forming a clear isolation‐by‐distance spatial pattern: Salticidae and Thomisidae. Caucasian populations of most of these spiders are effectively isolated from conspecific populations in Central Europe and less likely had gene exchange with those at least since the Holocene. Those are spiders effectively using plant surface and catching prey from ambush. Finally, (3) most ground‐dwelling Gnaphosidae and Lycosidae and vegetation‐dwelling Clubionidae are effectively isolated from the conspecific populations in Central Europe, at least since the Last Glacial Maximum, but in many ground spiders since even pre‐Pleistocene; however, within the region of the Caucasus and Asia Minor, most of wolf and sac spiders do not form isolation‐by‐distance spatial structure, suggesting their high dispersal potential and cohesive populations within this geographic region.

We described a general pattern; however, there are many exceptions that require individual explanations. Three species of actively hunting spiders, *Piratula latitans* (Lycosidae), *Pellenes nigrociliatus* (Salticidae), and *X. acerbus* (Thomisidae), share mitochondrial haplotypes between the Caucasus and Central Europe, *C. pallulida* (Clubionidae), and *T. terricola* (Lycosidae) genetically identical to the Caucasian individual is found even in Canada. Besides, three species of Thomisidae, two Salticidae, one Lycosidae, and one Gnaphosidae differ with only a single mutation from the conspecific spiders in Central or Northern Europe. A potential explanation for this may be the recent expansion of these species associated with human activities. Whereas in the case of spiders from North America, the presence of individuals genetically identical or very close to the Caucasian and European ones is obviously a result of recent non‐desperate transportation by humans, the situation with the species that are identical in the Caucasus and Europe is less obvious, although a high proportion of alien species in local spider faunas (e.g., Logunov, 2024) suggest that human‐driven dispersal is quite likely. In any event, their genetic closeness is probably a result of gene exchange in a relatively recent geological time, perhaps in Holocene, when human activities, first of all agriculture, substantially transformed the landscape (Racimo et al., 2020).

Our study reveals the presence of some cryptic species among the studied spiders. In particular, *Certidia prominens* (Araneidae) shows 98%–100% genetic similarity with the conspecific individuals from the Caucasus to Central Europe; however, there are three sequences from Germany, identified as *C. prominens*, with only 92.3% coincidence with all other specimens, including those from the same country. This pattern results either from misidentification or the presence of two cryptic species in Central Europe. The sequences of *Argiope* cf. *lobata* and *Levellius caspicus* (Araneidae) from Pakistan show striking differences from both Caucasian and European ones, driving us to the conclusion that in the Central Asia, there are long‐isolated lineages, possibly different evolutionary species. The same is true for *Dipoena* cf. *torva* from the Iberian Peninsula. There are no genetic differences between *Arctosa* cf. *tbilisiensis* from the Caucasus and Iran, but the genetic differences between the populations from the Caucasus and Anatolia exceed 5% of the CO1 structure. One sequence of *Gnaphosa lucifuga* from Turkey strongly differs from multiple sequences from Georgia and Europe. One sequence of *H. silvestris* from Greece differs by 5.5% from the conspecific sequences from Georgia to Germany. The same is true for *Psammitis ninii* from Spain. There are no differences between sequences of *Tiso vagans* from Switzerland and *Heterotheridion nigrovariegatum* from Slovenia; simultaneously, the differences between these two and the rest of conspecific sequences, from Georgian and Belarus to Germany, exceed 5%. In some cases, this may result from incorrect species identification; however, one can expect that this modest study, covering only 124 species of Georgian spiders from eight families, may help identifying the presence of up to 10 new cryptic species from different parts of Eurasia.

In their recent article, Anderson and Weir (2022) showed that, at least in vertebrates, the divergent ecological adaptation is not a common consequence of allopatric speciation; in most of the studied groups, adaptive divergence did not significantly increase in geographically isolated, closely related species. So far, the pattern is less clear for invertebrate animals, especially arthropods. Arthropods, including insects and arachnids, are much more speciose than vertebrate animals. There are over 51,900 described species of spiders (order Araneae) worldwide (WSC, 2024), which is more than all terrestrial vertebrates, comprising <35,000 species (Wiens, 2015). The vast majority of these species are described based solely on phenotypic characters, which drives us to conclusion that the morphological divergence in this group is much faster than in vertebrates, including divergence of allopatric evolutionary lineages. However, presence of strongly deviated sequences within the same nominal species suggests that cryptic speciation in spiders with no clear phenotypic divergence is quite common.

Our study suggests that large‐scale barcoding projects can help for deeper insight into the ecological and evolutionary patterns in diverse taxonomic groups, a synthetic analysis that is difficult with traditional taxonomic methods or genetic studies of individual evolutionary lineages. In our case, the analysis of 3320 sequences from different parts of the World, which belong to 124 spider species, obtained within the ongoing project and downloaded from the BOLD database, reveals several important patterns. (1) Most of the spider families show clear isolation‐by‐distance patterns within western Eurasia and form cohesive geographic populations throughout this region; (2) the dispersal ability of spiders is directly related to the web building and ballooning and significantly differs between the families of ambushing and active predators; other ways of expansion are less important in the formation of regional spider faunas; (3) ground‐dwelling spiders have a lower ability of dispersal to large distances than plant‐dwelling ones; (4) high dispersal ability not only increases the effective population size but also alpha diversity of spiders, both on species and genus level; and (5) there is up to 10% of spiders in local faunas, which expanded their ranges by human‐associated dispersal. Besides, large barcoding projects assist taxonomists in identifying cryptic evolutionary lineages from individual small regions.

And finally, it is important to understand that the studied fragment of CO1 gene gives limited information about genetic differentiation, although provides a chance to analyze simultaneously representative samples of major taxonomic groups. The next challenge is to establish a more precise time frame for the supposed gene flow between the geographically distant populations. This challenge can be solved using genomic methodologies, such as dRAD sequencing, which proved to be a powerful tool in spider species delimitation in some spider groups (Ivanov et al., 2021).

## AUTHOR CONTRIBUTIONS

**David Tarkhnishvili:** Conceptualization (lead); formal analysis (lead); investigation (equal); methodology (equal); project administration (supporting); resources (supporting); supervision (supporting); writing – original draft (lead); writing – review and editing (equal). **Armen Seropian:** Data curation (lead); investigation (supporting); methodology (supporting); validation (lead); writing – review and editing (supporting). **Christoph Erhardt:** Software (lead); writing – review and editing (supporting). **Nino Kachlishvili:** Data curation (supporting); investigation (lead). **Hans‐Joachim Krammer:** Data curation (equal); validation (supporting); writing – review and editing (supporting). **Nils Hein:** Conceptualization (supporting); funding acquisition (lead); methodology (supporting); project administration (lead); resources (supporting); supervision (equal); writing – review and editing (supporting).

## CONFLICT OF INTEREST STATEMENT

None declared.

## Supporting information


Table S1.

Table S2.

## Figures and Tables

**FIGURE A1:**
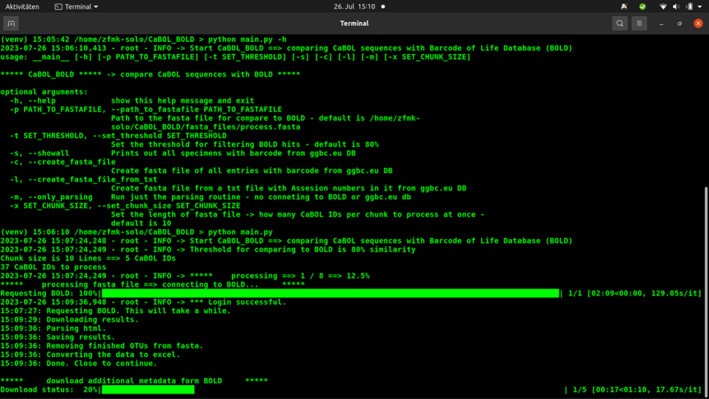
Connecting to BOLD and download data. The execute argument options are also visible on top. The prompt command “python main.py ‐h” will show this options.

**FIGURE A2:**
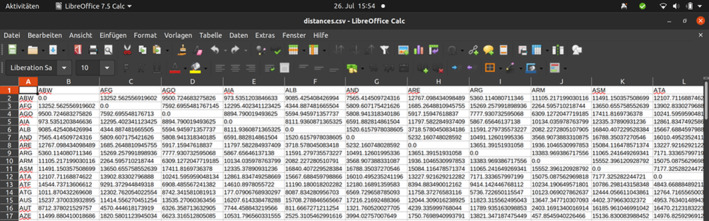
Part of the table with distances between countries.

**FIGURE A3:**
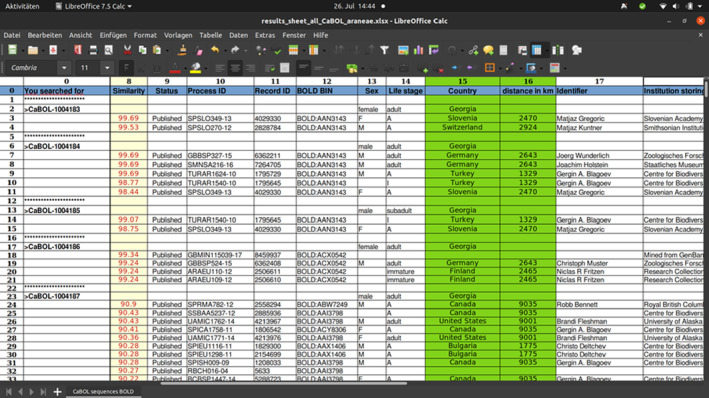
Part of the final Excel result sheet with similarity rate and distances showing.

## Data Availability

All data generated or analyzed during this study are included in this article (and its Tables S1 and S2 and Appendices 1 and 2). Any additional information related to this study is available from the corresponding author on request.

## APPENDIX 1

CaBOL_BOLD program developed by Christoph Erhardt.

The python program CaBOL_BOLD is automatically comparing a fasta file with the online Barcode of Life Database (BOLD) and downloads the hits and the associated metadata. Then, the distance between source countries from this metadata and the CaBOL specimen source country is calculated automatically and written into an preformatted Excel result sheet alongside all the metadata for further scientific analysis.

The program is divided into two parts—the download routine and the calculating/refactoring routine.

### Download routine

The given fasta file consisting of the CaBOL ID's and corresponding CO1 barcode sequences is split into chunks. This chunks will be compared with BOLD CO1 barcode sequences one by one. The chunk size is by default 5 CaBOL ID's and can be changed over an execute argument.

For the connection to BOLD and comparing the CaBOL ID's with BOLD database entries the program uses the Python package BOLDigger (Buchner & Leese, 2020; Figure A1).

BOLD gives back the TOP20 hits. Only the hits with public status can be evaluated because the source country data entry is given in the hits metadata. The raw results from BOLD are saved and extracted to a CSV file for further processing.

### Calculating/refactoring routine

After the download procedure, the dataset is filtered for nonpublic status and similarity. Default similarity threshold is 80% but can be changed with a startup argument (e.g., python main.py ‐t 95).

Afterward, the source country of the BOLD hits is extracted from the BOLD results CSV file and converted to ISO 3166‐1 alpha‐3 country code. For this task, a CSV file with the names of the countries and the corresponding ISO 3166‐1 alpha‐3 code is searched.

With this 3‐letter country code, a table with the distances between all the countries of the world in ISO 3166‐1 alpha‐3 format is then referenced (Figures A2 and A3).

The resulting distances in kilometer for every CaBOL ID and the BOLD hits—as well as the similarity rate between the given sample and the BOLD hit, the direct link to the BOLD specimen details page, and other metadata from CaBOL database and BOLD database useful to this article—is refactored and saved into the Excel result sheet.

The calculating/refactoring routine can be started separately from the download routine via an execute argument so the raw data from BOLD can be refactored without downloading again.

A detailed setup manual and instructions how to use the program is available in a readme file. The python program CaBOL_BOLD and all associated files can be download from GitLab https://gitlab.leibniz‐lib.de/cabol/cabol_bold.

## APPENDIX 2

The model of molecular divergence based on the assumption of the Poisson distribution of the substitution number dependent on a time passed.

In each cell of the table, probability is shown that a given number of substitutions occurs within a given time period, based on the assumption of Poisson distribution of the substitution number.

Kya (1st row) – thousands of years; Column C: the number of substitutions per studied fragment. Probabilities for each column making up the value exceeding 0.95 are marked in yellow color. E.g., For 10 Kya, cumulative probability of 0 and 1 substitution is 0.996 > 0.95; for 900 Kya, cumulative probability of 3 to 13 substitutions is 0.957 > 0.95.

**Table:**

Molecular clock for insects (Reference)	https://pubmed.ncbi.nlm.nih.gov/20167609/
Average proportion of substitutions in CO1 per year (Papadopoulou et al., 2010)	0.000000013
Studied CO1 fragment length (bp)	658
Average expected number of substitutions in the studied fragment per 100,000 years	0.8554

		Kya	10	20	30	40	50	60	70	80	90	100	200	300	400	500	600	700	800	900	1000	1100
			0.1	0.2	0.3	0.4	0.5	0.6	0.7	0.8	0.9	1	2	3	4	5	6	7	8	9	10	11
Average_mutations/100,000_years	0.8554	0	0.918016	0.842754	0.773662	0.710235	0.652007	0.598553	0.549482	0.504433	0.463078	0.425113	0.180721	0.076827	0.03266	0.013884	0.005902	0.002509	0.001067	0.000453	0.000193	8.2E‐05
		1	0.078527	0.144178	0.198537	0.243014	0.278863	0.307201	0.329019	0.345194	0.356505	0.363642	0.309178	0.197153	0.11175	0.059383	0.030293	0.015024	0.0073	0.003491	0.001649	0.000771
	Substitutions number	2	0.003359	0.012333	0.025474	0.041575	0.059635	0.078834	0.098505	0.118111	0.13723	0.15553	0.264471	0.252967	0.191182	0.12699	0.077739	0.044982	0.024976	0.013438	0.007053	0.003628
		3	9.58E‐05	0.000703	0.002179	0.004742	0.008502	0.013487	0.019661	0.026942	0.035216	0.044347	0.150819	0.216388	0.218049	0.181046	0.132996	0.089781	0.056972	0.034485	0.02011	0.011378
		4	2.05E‐06	3.01E‐05	0.00014	0.000406	0.000909	0.001731	0.002943	0.004609	0.006778	0.009484	0.064505	0.138824	0.186519	0.193583	0.170647	0.134397	0.097468	0.066371	0.043004	0.026766
		5	3.5E‐08	1.03E‐06	7.18E‐06	2.78E‐05	7.78E‐05	0.000178	0.000352	0.000631	0.001044	0.001622	0.022071	0.07125	0.127639	0.165591	0.175165	0.160948	0.133398	0.102192	0.073572	0.050371
		6	4.99E‐10	2.93E‐08	3.07E‐07	1.58E‐06	5.54E‐06	1.52E‐05	3.52E‐05	7.19E‐05	0.000134	0.000231	0.006293	0.030474	0.072788	0.118039	0.149836	0.160621	0.152145	0.131123	0.104889	0.078993
		7	6.1E‐12	7.17E‐10	1.13E‐08	7.74E‐08	3.39E‐07	1.11E‐06	3.01E‐06	7.03E‐06	1.47E‐05	2.83E‐05	0.001538	0.011172	0.035579	0.072122	0.10986	0.137395	0.148737	0.144209	0.128174	0.106182
		8	6.53E‐14	1.53E‐11	3.61E‐10	3.31E‐09	1.81E‐08	7.15E‐08	2.25E‐07	6.02E‐07	1.42E‐06	3.02E‐06	0.000329	0.003584	0.015217	0.038558	0.070481	0.102837	0.12723	0.138776	0.13705	0.124889
		9	6.2E‐16	2.92E‐13	1.03E‐11	1.26E‐10	8.6E‐10	4.08E‐09	1.5E‐08	4.57E‐08	1.21E‐07	2.87E‐07	6.25E‐05	0.001022	0.005785	0.018324	0.040193	0.068419	0.09674	0.118709	0.130258	0.13057
		10	5.31E‐18	4.99E‐15	2.64E‐13	4.3E‐12	3.68E‐11	2.09E‐10	8.97E‐10	3.13E‐09	9.33E‐09	2.46E‐08	1.07E‐05	0.000262	0.001979	0.007837	0.020629	0.040968	0.066201	0.091389	0.111423	0.122859
		11	4.13E‐20	7.76E‐17	6.16E‐15	1.34E‐13	1.43E‐12	9.76E‐12	4.88E‐11	1.95E‐10	6.53E‐10	1.91E‐09	1.66E‐06	6.12E‐05	0.000616	0.003047	0.009625	0.022301	0.041184	0.063961	0.086646	0.105093
		12	2.94E‐22	1.11E‐18	1.32E‐16	3.82E‐15	5.1E‐14	4.17E‐13	2.44E‐12	1.11E‐11	4.19E‐11	1.36E‐10	2.37E‐07	1.31E‐05	0.000176	0.001086	0.004117	0.011128	0.023486	0.041034	0.061765	0.082405
		13	1.94E‐24	1.46E‐20	2.6E‐18	1E‐16	1.68E‐15	1.65E‐14	1.12E‐13	5.85E‐13	2.48E‐12	8.96E‐12	3.12E‐08	2.58E‐06	4.62E‐05	0.000357	0.001625	0.005125	0.012363	0.0243	0.040641	0.059645
		14	1.18E‐26	1.78E‐22	4.77E‐20	2.46E‐18	5.13E‐17	6.04E‐16	4.8E‐15	2.86E‐14	1.36E‐13	5.48E‐13	3.81E‐09	4.73E‐07	1.13E‐05	0.000109	0.000596	0.002192	0.006043	0.013363	0.024832	0.040087
		15	6.74E‐29	2.03E‐24	8.15E‐22	5.6E‐20	1.46E‐18	2.07E‐17	1.92E‐16	1.3E‐15	7E‐15	3.12E‐14	4.35E‐10	8.1E‐08	2.58E‐06	3.11E‐05	0.000204	0.000875	0.002757	0.006858	0.014161	0.025147
		16	3.61E‐31	2.17E‐26	1.31E‐23	1.2E‐21	3.91E‐20	6.63E‐19	7.17E‐18	5.58E‐17	3.37E‐16	1.67E‐15	4.65E‐11	1.3E‐08	5.51E‐07	8.32E‐06	6.54E‐05	0.000327	0.001179	0.0033	0.007571	0.014788
		17	1.81E‐33	2.18E‐28	1.97E‐25	2.41E‐23	9.83E‐22	2E‐20	2.53E‐19	2.24E‐18	1.53E‐17	8.4E‐17	4.68E‐12	1.96E‐09	1.11E‐07	2.09E‐06	1.97E‐05	0.000115	0.000475	0.001494	0.003809	0.008185
		18	8.62E‐36	2.07E‐30	2.81E‐27	4.58E‐25	2.34E‐23	5.71E‐22	8.4E‐21	8.53E‐20	6.53E‐19	3.99E‐18	4.45E‐13	2.8E‐10	2.11E‐08	4.97E‐07	5.63E‐06	3.84E‐05	0.00018	0.000639	0.00181	0.004279
		19	3.88E‐38	1.87E‐32	3.8E‐29	8.25E‐27	5.26E‐25	1.54E‐23	2.65E‐22	3.07E‐21	2.64E‐20	1.8E‐19	4.01E‐14	3.78E‐11	3.8E‐09	1.12E‐07	1.52E‐06	1.21E‐05	6.5E‐05	0.000259	0.000815	0.002119
		20	1.66E‐40	1.6E‐34	4.88E‐31	1.41E‐28	1.12E‐26	3.96E‐25	7.93E‐24	1.05E‐22	1.02E‐21	7.69E‐21	3.43E‐15	4.84E‐12	6.49E‐10	2.39E‐08	3.9E‐07	3.62E‐06	2.22E‐05	9.97E‐05	0.000349	0.000997
		21	6.76E‐43	1.3E‐36	5.96E‐33	2.3E‐30	2.29E‐28	9.67E‐27	2.26E‐25	3.43E‐24	3.73E‐23	3.13E‐22	2.79E‐16	5.92E‐13	1.06E‐10	4.88E‐09	9.54E‐08	1.03E‐06	7.25E‐06	3.65E‐05	0.000142	0.000447
		22	2.63E‐45	1.01E‐38	6.95E‐35	3.58E‐32	4.45E‐30	2.26E‐28	6.15E‐27	1.07E‐25	1.31E‐24	1.22E‐23	2.17E‐17	6.9E‐14	1.65E‐11	9.48E‐10	2.22E‐08	2.81E‐07	2.25E‐06	1.28E‐05	5.52E‐05	0.000191
		23	9.78E‐48	7.53E‐41	7.76E‐37	5.32E‐34	8.28E‐32	5.03E‐30	1.6E‐28	3.17E‐27	4.37E‐26	4.53E‐25	1.61E‐18	7.7E‐15	2.45E‐12	1.76E‐10	4.96E‐09	7.31E‐08	6.71E‐07	4.28E‐06	2.05E‐05	7.82E‐05
		24	3.49E‐50	5.37E‐43	8.3E‐39	7.59E‐36	1.48E‐33	1.08E‐31	4E‐30	9.04E‐29	1.4E‐27	1.61E‐26	1.15E‐19	8.24E‐16	3.49E‐13	3.14E‐11	1.06E‐09	1.82E‐08	1.91E‐07	1.37E‐06	7.32E‐06	3.06E‐05
		25	1.19E‐52	3.67E‐45	8.51E‐41	1.04E‐37	2.52E‐35	2.21E‐33	9.57E‐32	2.48E‐30	4.32E‐29	5.52E‐28	7.88E‐21	8.46E‐17	4.78E‐14	5.37E‐12	2.18E‐10	4.37E‐09	5.23E‐08	4.23E‐07	2.5E‐06	1.15E‐05
		26	3.92E‐55	2.42E‐47	8.4E‐43	1.37E‐39	4.15E‐37	4.36E‐35	2.2E‐33	6.52E‐32	1.28E‐30	1.82E‐29	5.18E‐22	8.35E‐18	6.29E‐15	8.84E‐13	4.3E‐11	1.01E‐09	1.38E‐08	1.25E‐07	8.24E‐07	4.17E‐06
		27	1.24E‐57	1.53E‐49	7.99E‐45	1.73E‐41	6.58E‐39	8.29E‐37	4.89E‐35	1.65E‐33	3.65E‐32	5.76E‐31	3.28E‐23	7.93E‐19	7.97E‐16	1.4E‐13	8.18E‐12	2.23E‐10	3.49E‐09	3.57E‐08	2.61E‐07	1.45E‐06
		28	3.8E‐60	9.36E‐52	7.32E‐47	2.12E‐43	1E‐40	1.52E‐38	1.05E‐36	4.04E‐35	1E‐33	1.76E‐32	2.01E‐24	7.27E‐20	9.73E‐17	2.14E‐14	1.5E‐12	4.77E‐11	8.53E‐10	9.82E‐09	7.97E‐08	4.89E‐07
		29	1.12E‐62	5.52E‐54	6.48E‐49	2.5E‐45	1.48E‐42	2.69E‐40	2.16E‐38	9.52E‐37	2.66E‐35	5.19E‐34	1.18E‐25	6.43E‐21	1.15E‐17	3.16E‐15	2.65E‐13	9.86E‐12	2.01E‐10	2.61E‐09	2.35E‐08	1.59E‐07
		30	3.19E‐65	3.15E‐56	5.54E‐51	2.85E‐47	2.11E‐44	4.6E‐42	4.31E‐40	2.17E‐38	6.83E‐37	1.48E‐35	6.75E‐27	5.5E‐22	1.31E‐18	4.5E‐16	4.54E‐14	1.97E‐12	4.59E‐11	6.69E‐10	6.71E‐09	4.97E‐08
		31	8.81E‐68	1.74E‐58	4.59E‐53	3.14E‐49	2.91E‐46	7.62E‐44	8.32E‐42	4.8E‐40	1.7E‐38	4.08E‐37	3.73E‐28	4.56E‐23	1.45E‐19	6.21E‐17	7.52E‐15	3.8E‐13	1.01E‐11	1.66E‐10	1.85E‐09	1.51E‐08
		32	2.36E‐70	9.29E‐61	3.68E‐55	3.36E‐51	3.9E‐48	1.22E‐45	1.56E‐43	1.03E‐41	4.08E‐40	1.09E‐38	1.99E‐29	3.65E‐24	1.55E‐20	8.29E‐18	1.21E‐15	7.11E‐14	2.17E‐12	4E‐11	4.95E‐10	4.44E‐09
		33	6.11E‐73	4.81E‐63	2.86E‐57	3.49E‐53	5.05E‐50	1.9E‐47	2.83E‐45	2.13E‐43	9.52E‐42	2.83E‐40	1.03E‐30	2.84E‐25	1.6E‐21	1.08E‐18	1.87E‐16	1.29E‐14	4.5E‐13	9.32E‐12	1.28E‐10	1.27E‐09
		34	1.54E‐75	2.42E‐65	2.16E‐59	3.51E‐55	6.35E‐52	2.87E‐49	4.98E‐47	4.28E‐45	2.16E‐43	7.11E‐42	5.2E‐32	2.14E‐26	1.61E‐22	1.35E‐19	2.83E‐17	2.27E‐15	9.05E‐14	2.11E‐12	3.23E‐11	3.5E‐10
		35	3.75E‐78	1.18E‐67	1.58E‐61	3.43E‐57	7.76E‐54	4.21E‐51	8.51E‐49	8.37E‐47	4.74E‐45	1.74E‐43	2.54E‐33	1.57E‐27	1.58E‐23	1.65E‐20	4.15E‐18	3.89E‐16	1.77E‐14	4.64E‐13	7.88E‐12	9.42E‐11

		Kya	1200	1300	1400	1500	1600	1700	1800	1900	2000	2100	2200	2300	2400	2500	2600	2700	2800	2900	3000
			12	13	14	15	16	17	18	19	20	21	22	23	24	25	26	27	28	29	30
Average_mutations/100,000_years	0.8554	0	3.48E‐05	1.48E‐05	6.3E‐06	2.68E‐06	1.14E‐06	4.84E‐07	2.06E‐07	8.74E‐08	3.72E‐08	1.58E‐08	6.72E‐09	2.85E‐09	1.21E‐09	5.16E‐10	2.19E‐10	9.32E‐11	3.96E‐11	1.69E‐11	7.16E‐12
		1	0.000358	0.000165	7.54E‐05	3.43E‐05	1.56E‐05	7.03E‐06	3.17E‐06	1.42E‐06	6.36E‐07	2.84E‐07	1.26E‐07	5.62E‐08	2.49E‐08	1.1E‐08	4.88E‐09	2.15E‐09	9.49E‐10	4.18E‐10	1.84E‐10
	Substitutions number	2	0.001835	0.000916	0.000451	0.00022	0.000107	5.11E‐05	2.44E‐05	1.15E‐05	5.44E‐06	2.55E‐06	1.19E‐06	5.53E‐07	2.56E‐07	1.18E‐07	5.42E‐08	2.49E‐08	1.14E‐08	5.18E‐09	2.36E‐09
		3	0.00628	0.003394	0.001802	0.000942	0.000486	0.000248	0.000125	6.25E‐05	3.1E‐05	1.53E‐05	7.46E‐06	3.62E‐06	1.75E‐06	8.41E‐07	4.02E‐07	1.91E‐07	9.08E‐08	4.29E‐08	2.02E‐08
		4	0.016116	0.009436	0.005396	0.003023	0.001663	0.000901	0.000482	0.000254	0.000133	6.85E‐05	3.51E‐05	1.78E‐05	8.98E‐06	4.5E‐06	2.24E‐06	1.11E‐06	5.44E‐07	2.66E‐07	1.29E‐07
		5	0.033085	0.020987	0.012923	0.007757	0.004553	0.002621	0.001483	0.000826	0.000454	0.000246	0.000132	7.01E‐05	3.69E‐05	1.92E‐05	9.95E‐06	5.11E‐06	2.6E‐06	1.32E‐06	6.64E‐07
		6	0.056601	0.038896	0.025794	0.016588	0.010387	0.006353	0.003805	0.002238	0.001294	0.000737	0.000414	0.00023	0.000126	6.85E‐05	3.69E‐05	1.97E‐05	1.04E‐05	5.45E‐06	2.84E‐06
		7	0.083	0.06179	0.044128	0.030406	0.020308	0.013197	0.00837	0.005195	0.003163	0.001892	0.001114	0.000646	0.00037	0.000209	0.000117	6.49E‐05	3.56E‐05	1.93E‐05	1.04E‐05
		8	0.106497	0.085889	0.066057	0.048768	0.034743	0.023988	0.01611	0.010555	0.006763	0.004248	0.00262	0.001589	0.00095	0.00056	0.000326	0.000187	0.000106	5.99E‐05	3.34E‐05
		9	0.121463	0.106123	0.087897	0.069526	0.052834	0.038759	0.027561	0.01906	0.012856	0.008479	0.005479	0.003475	0.002167	0.00133	0.000805	0.00048	0.000283	0.000165	9.53E‐05
		10	0.12468	0.118011	0.105262	0.089209	0.07231	0.056363	0.042436	0.030978	0.021995	0.015231	0.01031	0.006836	0.004448	0.002844	0.00179	0.00111	0.000679	0.00041	0.000244
		11	0.116347	0.1193	0.114598	0.104058	0.08997	0.074511	0.0594	0.04577	0.034208	0.024872	0.017638	0.012227	0.008301	0.005529	0.003619	0.00233	0.001478	0.000924	0.00057
		12	0.099523	0.110554	0.114365	0.111264	0.102614	0.090294	0.076216	0.06199	0.048769	0.037232	0.027661	0.020046	0.014202	0.009853	0.006706	0.004484	0.002949	0.00191	0.00122
		13	0.078583	0.094568	0.105353	0.109818	0.108032	0.101003	0.09027	0.0775	0.06418	0.051448	0.040042	0.030338	0.022427	0.016209	0.011473	0.007967	0.005434	0.003645	0.002408
		14	0.057617	0.075115	0.090119	0.100648	0.105612	0.104911	0.099279	0.08997	0.078428	0.066012	0.053824	0.042634	0.032887	0.024759	0.018227	0.013142	0.009296	0.006459	0.004414
		15	0.039429	0.055686	0.071949	0.086095	0.096363	0.101707	0.101908	0.097483	0.08945	0.079054	0.067527	0.055919	0.045011	0.035298	0.027024	0.020236	0.014843	0.010682	0.007551
		16	0.025295	0.038703	0.053852	0.069042	0.082429	0.092437	0.098068	0.099022	0.095644	0.088755	0.079424	0.06876	0.057753	0.047178	0.037565	0.02921	0.02222	0.016561	0.012111
		17	0.015274	0.025317	0.037936	0.052111	0.066362	0.079071	0.088822	0.094668	0.096252	0.093785	0.087921	0.079577	0.069744	0.059347	0.049144	0.039684	0.031306	0.024166	0.018281
		18	0.00871	0.01564	0.025239	0.037146	0.050459	0.06388	0.075979	0.085478	0.091482	0.093594	0.091921	0.086978	0.079546	0.070508	0.060722	0.050918	0.041656	0.033304	0.026063
		19	0.004706	0.009154	0.015908	0.025085	0.036347	0.048891	0.061571	0.073118	0.082372	0.088488	0.091044	0.090064	0.085949	0.079359	0.071077	0.061895	0.052511	0.043482	0.035201
		20	0.002415	0.00509	0.009525	0.016094	0.024873	0.035548	0.047401	0.059418	0.070461	0.079477	0.085667	0.088597	0.088225	0.084854	0.07904	0.071475	0.062885	0.053933	0.045167
		21	0.001181	0.002695	0.005432	0.009833	0.016211	0.024616	0.034755	0.045986	0.057402	0.067985	0.076769	0.083004	0.086249	0.08641	0.083708	0.078609	0.071722	0.063709	0.055194
		22	0.000551	0.001362	0.002957	0.005735	0.010085	0.016271	0.024324	0.033972	0.044638	0.055511	0.065668	0.074229	0.080484	0.083994	0.084623	0.082524	0.078084	0.071836	0.064381
		23	0.000246	0.000659	0.00154	0.003199	0.006001	0.010287	0.016283	0.024006	0.033203	0.043355	0.05373	0.063495	0.07184	0.078096	0.081828	0.082868	0.081313	0.077479	0.071833
		24	0.000105	0.000305	0.000768	0.00171	0.003422	0.006233	0.010447	0.016257	0.023668	0.03245	0.042131	0.052051	0.061452	0.069587	0.075829	0.079746	0.081147	0.080083	0.076807
		25	4.32E‐05	0.000136	0.000368	0.000878	0.001874	0.003626	0.006434	0.010568	0.016197	0.023316	0.031714	0.040962	0.050463	0.059525	0.067458	0.073672	0.077743	0.079463	0.078841
		26	1.7E‐05	5.81E‐05	0.00017	0.000433	0.000986	0.002028	0.00381	0.006606	0.010657	0.016109	0.022955	0.030996	0.039846	0.048959	0.057704	0.065443	0.071617	0.075816	0.077816
		27	6.48E‐06	2.39E‐05	7.52E‐05	0.000206	0.0005	0.001092	0.002173	0.003977	0.006753	0.010718	0.015999	0.022586	0.030297	0.038777	0.047532	0.05598	0.06353	0.069657	0.07396
		28	2.38E‐06	9.5E‐06	3.22E‐05	9.43E‐05	0.000244	0.000567	0.001195	0.002308	0.004126	0.006876	0.010753	0.01587	0.022214	0.029616	0.037755	0.046175	0.054344	0.061712	0.067784
		29	8.41E‐07	3.64E‐06	1.33E‐05	4.17E‐05	0.000115	0.000284	0.000634	0.001294	0.002434	0.004259	0.006978	0.010767	0.015725	0.021839	0.028954	0.036774	0.044883	0.052789	0.059982
		30	2.88E‐07	1.35E‐06	5.3E‐06	1.79E‐05	5.26E‐05	0.000138	0.000326	0.000701	0.001388	0.00255	0.004377	0.007061	0.010761	0.015568	0.021465	0.028311	0.035833	0.04365	0.051309
		31	9.53E‐08	4.84E‐07	2.05E‐06	7.39E‐06	2.32E‐05	6.47E‐05	0.000162	0.000367	0.000766	0.001478	0.002657	0.004481	0.007127	0.010739	0.0154	0.021092	0.027685	0.03493	0.042474
		32	3.06E‐08	1.68E‐07	7.66E‐07	2.96E‐06	9.93E‐06	2.94E‐05	7.78E‐05	0.000187	0.00041	0.00083	0.001563	0.002755	0.004572	0.007177	0.010703	0.015223	0.020722	0.027078	0.034061
		33	9.5E‐09	5.67E‐08	2.78E‐07	1.15E‐06	4.12E‐06	1.3E‐05	3.63E‐05	9.19E‐05	0.000212	0.000452	0.000891	0.001643	0.002844	0.004651	0.007213	0.010654	0.01504	0.020355	0.026487
		34	2.87E‐09	1.85E‐08	9.8E‐08	4.35E‐07	1.66E‐06	5.54E‐06	1.64E‐05	4.39E‐05	0.000107	0.000239	0.000493	0.00095	0.001717	0.002925	0.004718	0.007237	0.010595	0.014851	0.019992
		35	8.42E‐10	5.89E‐09	3.35E‐08	1.59E‐07	6.49E‐07	2.3E‐06	7.23E‐06	2.04E‐05	5.22E‐05	0.000122	0.000265	0.000534	0.001007	0.001787	0.002998	0.004776	0.00725	0.010526	0.014658

14,400.00
14,400.00
7200.00
7200.00
5000
2100.00
4700
5000
60,000.00
